# Visualizing natural history collection data provides insight into collection development and bias

**DOI:** 10.3897/BDJ.6.e26741

**Published:** 2018-10-03

**Authors:** Vaughn Shirey

**Affiliations:** 1 Department of Entomology, The Academy of Natural Sciences of Drexel University, Philadelphia, United States of America Department of Entomology, The Academy of Natural Sciences of Drexel University Philadelphia United States of America; 2 Department of Biology, Georgetown University, Washington, D.C., United States of America Department of Biology, Georgetown University Washington, D.C. United States of America

**Keywords:** natural history collections, data bias, biodiversity informatics, visualization

## Abstract

Natural history collections contain estimated billions of records representing a large body of knowledge about the diversity and distribution of life on Earth. Assessments of various forms of bias within the aggregated data associated with specimens in these collections have been conducted across temporal, taxonomic, and spatial domains. Considering that these biases are the sum of biases across all contributing collections to aggregate datasets, the assessment of bias at the collection level is warranted. Interactive visualization provides a powerful tool for the assessment of these biases and insight into the historical development of natural history collections, providing context for where sources of bias may originate and developing historical narratives to clarify our understanding of our own knowledge about life on Earth. Here, I present a case study on using Sankey diagrams to illustrate the development of the entomology type collection at the Academy of Natural Sciences of Drexel University in Philadelphia, Pennsylvania with the hope that extensions of these practices among individual natural history collections are modified and adopted.

## Introduction

Natural history collections (NHCs) are often a component of both private and public institutions, harboring a massive amount of preserved records of life on Earth. A recent estimate of the number of NHCs specimens with valuable information on the diversity and distribution of species suggests that there are 1.23-1.97 billion units (a digitized record that may encompass a single specimen or lot) globally (Ariño 2010). In recent decades, the digitization and aggregation of specimen metadata has brought forth the development of regional and global initiatives such as the Global Biodiversity Information Facility (GBIF), which currently provides over 980,000,000 specimens records for public access (Global Biodiversity Information Facility 2018).

These publicly accessible records have been subject to some concern regarding the inevitable biases across time, space, and taxonomy, as these aggregate data are the sum of both specialized and generalized survey efforts across the collections that contribute to the whole. Recently discussed concerns with aggregate biodiversity data include over- and underrepresentation of taxonomic groups, temporal, and spatial biases (Troudet et al. 2017, Boakes et al. 2010, Oliveira et al. 2016, Daru et al. 2017). In addition, temporal bias is especially relevant due to the change of quality in NHCs data over time (specifically concerning taxonomic revisions, data curation, and ecological dynamics) and potential downstream impacts for analysis. (Tessarolo et al. 2017).

While these biases have been highlighted at the level of aggregate NHCs datasets, elucidating the degree of bias within individual NHCs has been underexplored. This area of research presents opportunities for NHCs curators, curatorial assistants, interns, and the public to better understand the applications and limitations of NHCs data on an institution-by-institution basis and provides key insight that is necessary for mapping the history of collections across their lifetime. In addition to these benefits, the potential for collaborative, interdisciplinary work across the digital humanities is greatly expanded by utilizing NHC data to address questions outside of the domain of life sciences. For example, historical research focusing on collection in developing nations throughout time and the contributions of women, indigenous, and minority groups to biodiversity science have yet to be explored in depth. These issues of potential future humanities and social science based research relate directly to the Nagoya Protocol (United Nations 2015), and the protection of traditional knowledge associated with biodiversity and its genetic resources. Understanding how natural history and biodiversity has been conducted in historical settings will further awareness of conscious, socially-responsible biodiversity science in the coming era.

In this case study, I aim to explore one, multifaceted visualization of NHCs data to provide insight into the history of the entomology collections at The Academy of Natural Sciences of Drexel University (ANSP), located in Philadelphia, Pennsylvania. As the oldest NHC sin the western hemisphere, ANSP collections provide the largest temporal context for understanding the development of NHCs in the New World.

## Objectives, concept, and approach

Sankey diagrams are flow diagrams that have long been used to visualize thermodynamic or energetic processes as well as financial data. They are straightforward to understand because each component size is proportional to the frequency of whatever metric is being traced. For this case, I have chosen to focus on the relationship between time (represented by year of collection), taxon (encapsulated by taxonomic order), and collector(s) in order to determine if specific individuals, or groups of individuals, throughout time have influenced taxonomic representation within the collection due to personal taxonomic bias/interests. At ANSP, verbal, anecdotal evidence of taxonomic shifts (largely from Hymenoptera to Orthoptera) through time within the collection were known; however, the extent and exact shift was never analyzed from either a statistical or visual standpoint. These shifts were largely attributed to the composition of people working within the collection at the time (e.g. individuals focusing on a favored taxon, or a taxon with significant funding for study). By formalizing when these shifts have occurred (and who contributed to them), we can better understand the history of our collection and how it may or may not be suited to answering certain biological questions.

By constructing the Sankey diagram in a interactive framework, namely by using the "shiny" package in R (Winston et al. 2017, R Core Team 2017), the ability to illustrate and filter the often complex series of flows by taxon and year facilitates ease of viewing and the ability to construct a historical narrative around the development of a particular collection. In practice, additional tiers could be added to this basic model to include geographic contexts; however, the addition of additional tiers often obfuscates the interpretation of the product and each tier should be selected on a case by case basis to fit the nature of the question being asked.

Although narrow is scope, this case study is broadly extensible to exploring other variables such as biogeographic regions, countries, or finer taxonomic resolutions. In addition, this approach is scalable to larger datasets, potentially including GBIF given that the number of parameters being assessed are not too large because of inherent limitations to webpage response times and the size of a given display. For example, mapping this diagram to individual collectors may cause issues; however, grouping collectors by nationality or organization may help to alleviate some of the computational strain associated with multiple individual entities.

## Methodology

Considering that the entomology collection at ANSP is not fully digitized, I have elected to use the type collection of nearly 13,000 types as a proxy for the main collection. Regardless of this choice, the methodology hereafter is broadly applicable to NHCs regardless of taxonomic focus and collection size

Type specimen data for all 13,000 records were obtained from the ANSP Entomology Type Database (ANSP Entomology Department 2018) and saved as a CSV. The data was then modulated, removing all fields that were not relevant to this case study. Unique pairs of taxon and collector(s) as well as taxon and year were extracted and the frequency of each unique combination across the entire dataset was calculated. Collection data without a listed year was removed. Collector(s) were not split into individuals, as individual collectors may have different collecting behavior when placed in a collecting pair or group. In addition, collectors with the same surname could not be disentangled given that many labels do not include a first name or initial.

I created an interactive Sankey diagram application using "shiny" along with the "alluvial" package (Bojanowski and Edwards 2016). These packages were chosen due to the relative ease of implementation with the goal that non-experts in R could readily understand and implement this approach; however, they do pose limitations to visualization, and additional packages exist that may fit other questions better (see the "GoogleVis" package for an interactive but comparable visualization Gesmann and de Castillo 2011). For visualization clarity, collectors who contributed fewer than 10 specimens per taxon were omitted for that pairing, in contrast collectors who contributed over 100 specimens for a taxon are highlighted to show importance. The code and modified data (as CSV) are available through GitHub at https://github.com/vmshirey/GVisShiny_dev/ under the directory App-1.

## Results

**Figure 1.**
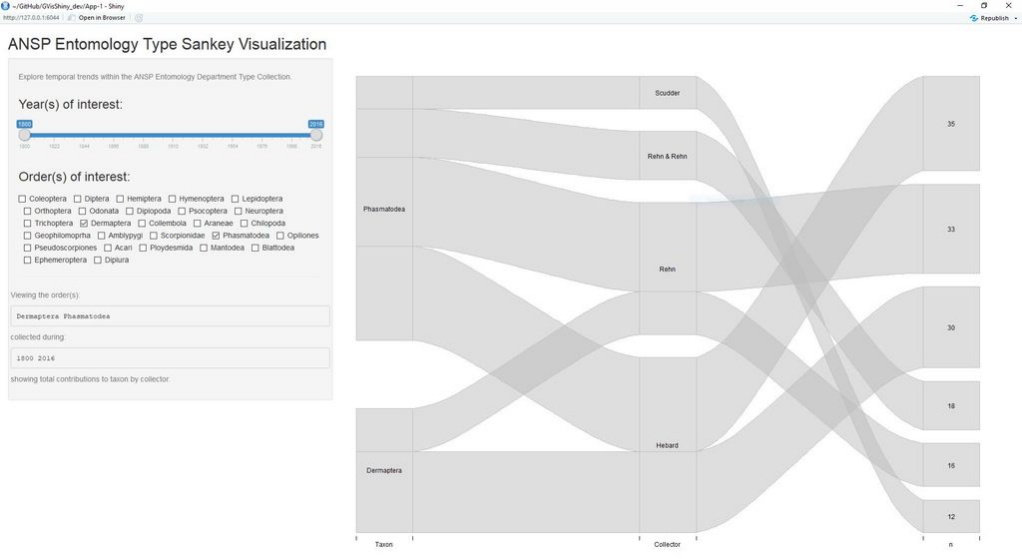
Application overview with interactive elements (left) and plot output (right). Note the display of selected taxa and collection years under the interactive elements for context

**Figure 2.**
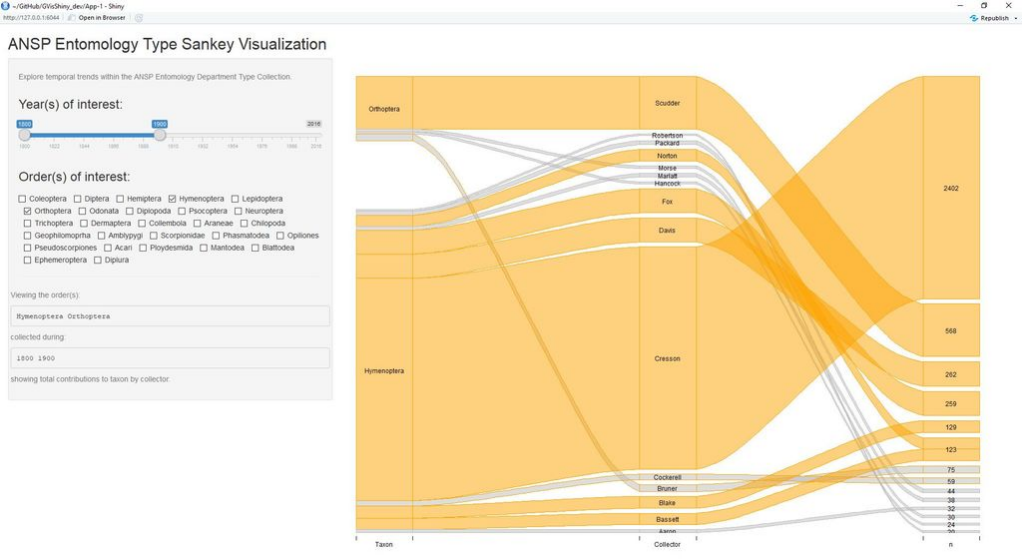
Collection of Hymenoptera and Orthoptera from the years 1800 through 1900. Hymenoptera is the larger order represented during this time period.

**Figure 3.**
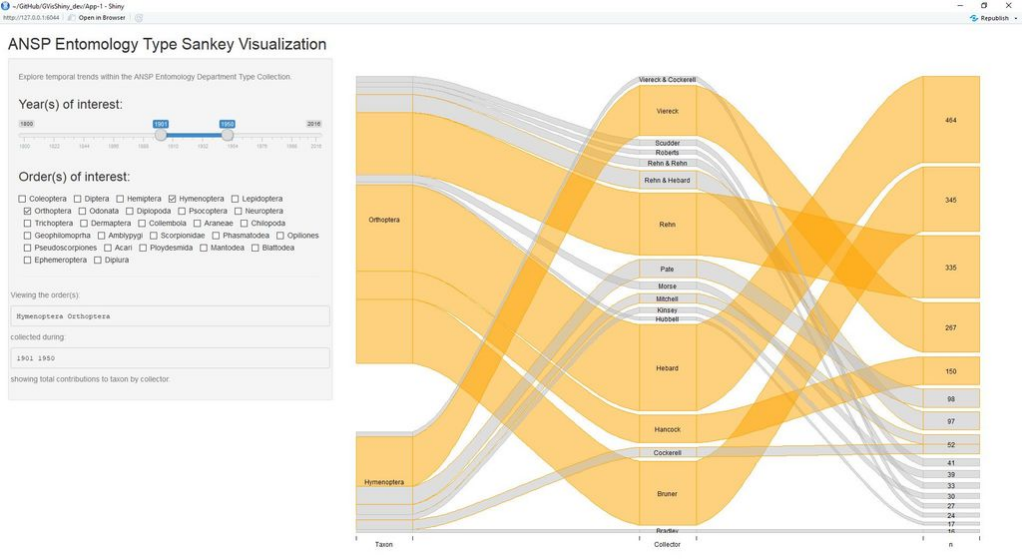
Collection of Hymenoptera and Orthoptera during the years 1901 through 1950. Here, Orthoptera additions take the lead over Hymenoptera records.

**Figure 4.**
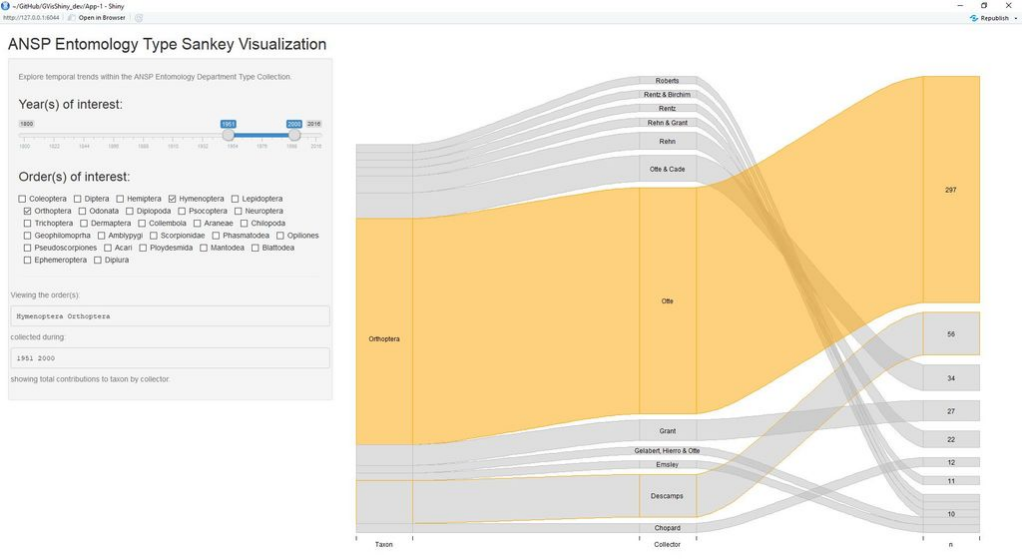
Collections of Hymenoptera and Orthoptera made from the years 1951 through 2000. Here, Hymenoptera additions are relatively non-existent, while Orthoptera additions are still numerous

The application alongside test case on anecdotal evidence suggesting a collection taxonomic composition turnover from Hymenoptera to Orthoptera is demonstrated through the following figures (Figs 1, 2, 3, 4). The output is ordered in three colums: (1) Taxon, (2) Collector, and (3) Number of Records.

These figures also elucidate additional information regarding collectors adding new specimens from particular groups. Among Hymenoptera and Orthoptera, relatively few collectors contribute to both taxa, instead focusing on a specific group (likely due to taxonomic interest/specific research).

## Conclusions

Visualization is a well/known, powerful tool for communicating science to a broad audience. Here, I have demonstrated the ability of Sankey diagrams in illustrating the developmental trends of an individual collection. Through additional interactivity, visualization frameworks become even more useful in generating historical collection narratives and addressing questions about how biases within specific NHCs arise. While this particular Sankey diagram represents just one possibility in employing interactive visualization within individual NHCs, there are a variety of additional visualization methods and platforms that do not require programming knowledge and exist as web-based tools. However, Sankey diagrams may facilitate easier understanding of data, especially relationships of multiple variables for a wide audience of collections specialists, scientists, and the general public.

Analysis of bias at multiple levels of data volume is beneficial across all domains of research that employ NHCs label data. Visualization provides multifaceted tools for individual institutions to not only understand the development of their own collections, but also articulate those stories to their stakeholders and through educational outreach. The development and accessibility of visualization methodologies for application across a wide variety of collections will further our understanding of collection development and give insight into which collections/consortiums can be targeted to fit certain research questions or for continued digitization (for example, in the case of underrepresented taxonomic groups, regions, or time frames). In addition, future visualization frameworks, when combined with demographic and other data from digital humanities, will unlock greater potential for understanding of our knowledge of life and Earth and how we got to this point in our understanding.
